# Promoter variations of *ClERF1* gene determines flesh firmness in watermelon

**DOI:** 10.1186/s12870-024-05000-z

**Published:** 2024-04-16

**Authors:** Yimei Zhou, Qinghui Shen, Lingmin Cai, Haoshun Zhao, Kejia Zhang, Yuyuan Ma, Yongming Bo, Xiaolong Lyu, Jinghua Yang, Zhongyuan Hu, Mingfang Zhang

**Affiliations:** 1 Laboratory of Germplasm Innovation and Molecular Breeding, College of Agriculture and Biotechnology, Zhejiang University, Hangzhou, China; 2 Zhejiang Engineering Research Center for Precision Crop Design Breeding, Hanghzou, China; 3 Ningbo Weimeng Seed Company, Ningbo, China; 4 Hainan Institute of Zhejiang University, Yazhou District, Sanya, China; 5 Key laboratory of Horticultural Plant growth, Development and Quality Improvement, Ministry of Agriculture, Hangzhou, China

**Keywords:** Watermelon, Flesh firmness, Fine mapping, *ClERF1*

## Abstract

**Background:**

Flesh firmness is a critical factor that influences fruit storability, shelf-life and consumer’s preference as well. However, less is known about the key genetic factors that are associated with flesh firmness in fresh fruits like watermelon.

**Results:**

In this study, through bulk segregant analysis (BSA-seq), we identified a quantitative trait locus (QTL) that influenced variations in flesh firmness among recombinant inbred lines (RIL) developed from cross between the *Citrullus mucosospermus* accession ZJU152 with hard-flesh and *Citrullus lanatus* accession ZJU163 with soft-flesh. Fine mapping and sequence variations analyses revealed that *ethylene-responsive factor 1* (*ClERF1*) was the most likely candidate gene for watermelon flesh firmness. Furthermore, several variations existed in the promoter region between C*lERF1* of two parents, and significantly higher expressions of C*lERF1* were found in hard-flesh ZJU152 compared with soft-flesh ZJU163 at key developmental stages. DUAL-LUC and GUS assays suggested much stronger promoter activity in ZJU152 over ZJU163. In addition, the kompetitive allele-specific PCR (KASP) genotyping datasets of RIL populations and germplasm accessions further supported C*lERF1* as a possible candidate gene for fruit flesh firmness variability and the hard-flesh genotype might only exist in wild species *C. mucosospermus*. Through yeast one-hybrid (Y1H) and dual luciferase assay, we found that ClERF1 could directly bind to the promoters of *auxin-responsive protein* (*ClAux/IAA*) and *exostosin family protein* (*ClEXT*) and positively regulated their expressions influencing fruit ripening and cell wall biosynthesis.

**Conclusions:**

Our results indicate that *ClERF1* encoding an ethylene-responsive factor 1 is associated with flesh firmness in watermelon and provide mechanistic insight into the regulation of flesh firmness, and the *ClERF1* gene is potentially applicable to the molecular improvement of fruit-flesh firmness by design breeding.

**Supplementary Information:**

The online version contains supplementary material available at 10.1186/s12870-024-05000-z.

## Background

Watermelon (*Citrullus lanatus*) is an economically important horticultural crop worldwide, which is favored by consumers because of its sweet and juicy taste, and nutritious value [1]. As a key factor affecting storability and shelf-life of watermelon fruit, flesh firmness would somehow determine the taste and subsequent consumer preference as well. If the flesh is too soft, it is easier to cause hollow defects with a shorter shelf life [2]. Although the hard flesh is less juicy and tasty [3], hard-flesh trait is especially desirable for its better transportability and longer shelf-life. In natural populations, flesh textures show great variability and become an important target trait in commercial breeding of watermelon.

The variation of flesh firmness is a complex process, including the changes of plant hormone and cell wall contents and related enzymes activities, which may be controlled by multiple genes [4–9]. Fruit softening process is related to changes in the components of the cell wall. In apple development, the decrease in fruit firmness was directly related to the reduction of cellulose, hemicellulose and soluble pectin [10]. Besides, cellulose content was discovered to be related with fruit firmness at the development stage of peach [11]. The genetic factors that control fruit firmness has also been reported in several fruit crops. The QTL responsible for tomato fruit firmness was located on chromosome 2, and an ethylene responsive factor and three pectin methylesterase genes were nominated as QTL candidate genes [12]. A study using the whole-genome sequencing data of the RILs identified a QTL (*qFIS1*) for fruit firmness in tomato, and finally demonstrated that *FIS1* encoding a GA2-oxidase regulates fruit firmness [13]. Using the genetic linkage map and GWAS, it was determined that the *ERF4* gene affected fruit firmness of apple [14]. In melon, two QTL (QTL*ff5.1* and QTL*ff2.1*) associated with fruit firmness were identified by using specific-locus amplified fragment sequencing with BSA [15]. The GWAS results of fruit firmness in *Pyrus* revealed that an identical-by-descent segment harboring a 12 bp insertion in *TIC55* determined fruit softening [16]. After the release of watermelon genome [17, 18], functional genomic studies for important quality and storability relevant traits are also facilitated [19–22]. *ClERF4* gene influencing fruit rind hardness and cracking was fine-mapped through BSA-seq in watermelon [20]. Linkage mapping and comparative transcriptome analysis of flesh firmness in watermelon revealed that the major genes controlling center flesh firmness were located on chromosome 2 and chromosome 8, and *Cla016033* (DUF579 family member) and *Cla012507* (MADS-box transcription factor) may respectively influence the cell wall contents and fruit ripening to affect the hardness of watermelon fruit [23]. Combine BSA-Seq and comparative transcriptomic revealed that a candidate gene *AUX/IAA* was related to the flesh firmness trait of watermelon [24]. In addition, *Aux/IAA* was also identified as a candidate gene associated with flesh firmness in watermelon through GWAS and BSR-seq [25].

The ERF proteins with an ERF DNA-binding domain can bind target promoters with cis-acting elements, such as a GCC box or a DRE (dehydration-responsive element, CCGAC), thereby regulating the transcription of these genes [26–30]. Many ERFs act as transcription factors to control the expression of some genes related to fruit ripening and cell wall biosynthesis. PpeERF2 was reported to represses the expression of cell wall related genes and ABA biosynthesis genes in peach ripening [31]. Besides, PpERF4 was found to enhance the transcription of *PpIAA1* gene by binding to its promoter to accelerate fruit ripening [32]. In persimmon (*Diospyros kaki L.*), DkERF18 activated *DkACS2* by binding its promoter, while DkERF8 and DkERF16 respectively bound to the promoter of *DkXTH11* and *DkEXP4* to increase their activities [33].

However, the key gene for flesh firmness influencing fruit palatability and storability remains largely unknown in watermelon. In this study, we developed RIL populations derived from the hard-flesh and soft-flesh hybrid. We used BSA-seq analysis to map the *flesh firmness* (*ClFF*) to chromosome 6. Through fine mapping and sequences analyses, we further revealed that *ethylene-responsive factor 1* (*ClERF1*) was the candidate gene responsible for flesh firmness. Through DNA-protein interaction analysis, we identified *ClAux/IAA* and *ClEXT* as the potential target genes of ClERF1 that were involved in regulation of fruit ripening and cell wall biosynthesis.

## Materials and methods

### Plant materials, trait evaluation and genetic analysis

We selected *Citrullus mucosospermus* accession ZJU152 with hard flesh and *Citrullus lanatus* accession ZJU163 with soft flesh as parent lines. The RIL populations were developed by hybridization between ZJU152 and ZJU163 and subsequent self-crossings. Seedlings of watermelon were raised in a greenhouse in Hangzhou City, China, in the spring of 2021 (germplasm accessions), 2022 (parents and F_6_ population) and 2023 (recombinant plants, F_8_ population and wild accessions) and in the autumn of 2022 (parents and some germplasm accessions). For the RIL populations, inbred lines were grown with four plants per line, and each plant is only allowed to bear one fruit. Field experiments were arranged in randomized complete blocks with ten inbred lines and four repetitions per block. We harvested 144 F_6_ inbred lines fruits in 2022 and 135 F_8_ inbred lines fruits in 2023. For parental materials, at four key developmental stages (10 DAP, 18 DAP, 26 DAP, 34 DAP), fruits were selected for firmness test and fruit fleshes were stored at -80 ℃ for further analyses. According to previous report, phenotypic and metabolic changes in watermelon were more prominent during these four key developmental stages between cultivated and wild watermelon [34]. Mature fruits were harvested at 40 days after pollination (DAP) for firmness determination. There were three biological replications for each sample of parents, F_1_ and RIL inbred lines. Mean differences in flesh firmness between two parents and F_1_were analyzed using paired Student’s t tests.

The firmness of fruit flesh was measured by a Texture Analyzer TA. XT- 21 (Stable Micro Systems Ltd., Godalming, Surrey, UK) with a p-7.5 probe. Three different points from center region and edge region of each fruit were selected to measure firmness index. Genetic analysis was performed using R software package SEA v2.0.1. Akaike’s information criterion (AIC) value was calculated in each model to identify the existence of major genes affecting quantitative traits [35].

### DNA extraction, quality detection and library construction

The genome DNA was extracted from fresh leaves of young seedlings using the cetyltrimethylammonium bromide (CTAB) procedure [36]. DNA quality and quantity were determined by agarose gel electrophoresis and Nanodrop 2000 (Thermo Fisher Scientific, USA). The Hard-pool and Soft-pool were constructed by mixing 20 hard-flesh and 20 soft-flesh samples from RIL individuals evenly. Truseq Nano DNA HT Sample preparation Kit (Illumina) was used to generate sequencing libraries, and the Illumina HiSeq PE150 platform was used to sequence these libraries. The quality of the sequencing data was determined using FASTQC [37].

### BSA-seq analysis

For sequence alignment, the genome of *Citrullus lanatus* (Watermelon (97,103) v2) (http://cucurbitgenomics.org/organism/21) [38] was used as a reference genome. Alignment files were converted to BAM files by SAMtools software [39, 40]. The Unified Genotype function and the Variant Filtration in GATK software were used to call single nucleotide polymorphism [41], and ANNOVAR software was used to annotate SNPs [42]. The homozygous SNPs between two parents (ZJU152 and ZJU163) were extracted from the VCF files. The SNP indexes were calculated based on the read depth information for homozygous SNPs [43] in the two offspring pools (Hard-pool and Soft-pool). The genotype of one parent was used as the reference and the statistic reads number of this reference parent in the offspring pool was calculated. The SNP indexes of base sites were then calculated by the ratio of different reads in the total number. We filtered out the points whose SNP indexes were less than 0.3 in both pools. Sliding window methods using a window size of 1 Mb and a step of 10 kb as the default setting, were employed to calculated the SNP indexes of the whole genome. The difference in the SNP index between two offspring pools was calculated as the ΔSNP index. The screening thresholds was chosen with a 99% confidence level. The candidate region for the target trait was identified as the peak region of ΔSNP index that was over the threshold.

### Haplotype analysis and fine mapping

Haplotype analysis was conducted for fine mapping and QTL validation in 144 F_6_ individuals and 135 F_8_ individuals, and was also done to determine the allelic variation in 126 watermelon germplasm accessions and 67 wild accessions. To narrow down the candidate region of initial mapping, we employed 17 pair of KASP primer combinations (Fam, Hex, R) as markers for genotyping in RIL population (Table S9). The details of KASP assay were as described in the literature [20]. After the amplification, the fluorescence signals were detected and the genotyping results were derived by LGC genomics system (Hoddesdon, UK).

### Cloning and qRT-PCR analysis of the candidate gene

The candidate gene was amplified from the DNA of two parents (ZJU152 and ZJU163) using the primers which were designed at NCBI website (Table S10). KOD ONE PCR Master Mix (Toyobo, Japan) was used for PCR amplification. The resulting PCR products were cloned into a pEASY-Blunt Zero Cloning Vector (TRANs, Beijing, China) according to the manufacturer’s instructions and amplified in *E coil* overnight. Clones were then sequenced by Zhejiang Youkang Biotechnology Co., Ltd. Sequence alignments were analyzed using SnapGene, and some clones whose sequence did not perfectly match the full-length reference were discarded. The qRT-PCR was employed to examine expression levels of candidate gene in the flesh of two parental lines at four key developmental stages and different tissues. The total RNAs of flesh and rind was isolated using Easy Plant (Polysaccharide and Polyphenols) RNA Extraction Kit (Easy-do, Hangzhou, China), and the total RNAs of stem, leaf, female flower and male flower were isolated using Easy RNA Extraction Kit (Easy-do, Hangzhou, China). The reactions of qRT-PCR were performed using the TOROGreen qPCR Master Mix (Toyobo, Japan), on an ABI Step One Plus system (Applied Biosystems, USA). The watermelon actin gene was used as an internal control in the analysis. All analyses were conducted with three biological and technical replications. All primers used for qRT-PCR are listed in Table S10.

### Transient GUS activity assay

Tobacco (*Nicotiana benthamiana*) leaves were used to conduct transient GUS activity assay [44]. The promoter sequences from ZJU152 and ZJU163 of *ClERF1* were cloned into pMDC162 vector to obtain GUS fusion expression vector using pEASY-Basic Seamless Cloning and Assembly Kit (TRANs, Beijing, China) according to the manufacturer’s instructions. The constructs were transformed into *Agrobacterium tumefaciens* GV3101 and then injected into 4-week-old tobacco leaves for Agrobacterium-mediated transformation. The CaMV35S-GUS vector was used as the positive control. The injected tobaccos were grown for two or three days under normal conditions, the tobacco leaves were collected for GUS staining and protein activity analysis. The instructions of the GUS staining Kit (SL7160), GUS Gene Quantitative Detection Kit (SL7161) and Bradford Protein Assay Kit (SK1060) can be referenced for details.

### Dual-luciferase assay

The promoter activity of *ClERF1* was examined using dual-luciferase assay [45]. The promoter sequences from ZJU152 and ZJU163 of *ClERF1* were cloned into pGreenII 0800-LUC reporter vectors upstream of the firefly luciferase (LUC) reporter gene. Besides, the pGreenII 0800-LUC reporter vectors also contained the renilla luciferase (REN) reporter gene which was driven by the CaMV 35S promoter. The plasmids were transformed into *Agrobacterium tumefaciens* GV3101 (pSoup) and injected the leaves of tobacco (*Nicotiana benthamiana*) for promoter activity examination. The full-length coding sequence of *ClERF1* was cloned into the pGreenII 62-SK vector to generate the 62-SK-ClERF1 effector vector, with the empty vector as a negative control. The promoters of *ClAux/IAA* and *ClEXT* was inserted into pGreenII 0800-LUC to generate the reporter vectors. These vectors were individually transformed into *Agrobacterium tumefaciens* GV3101 (pSoup), and the mixed bacterial solution of effector and reporters were injected into tobacco leaves using Agrobacterium-mediated transformation. Two to three days after injection, the tobacco leaves were harvested for LUC activity detection. Dual-Luciferase Reporter Gene Assay Kit (Yeasen, Shanghai, China) was used to measure the fluorescence values of LUC and REN.

### Measurement of cell wall components in watermelon flesh

The measurement of cell wall components (cellulose, hemicellulose and protopectin) was conducted to compare the contents of two parental lines at four developmental stages. According to the manufacturer’s instructions, cellulose, hemicellulose and protopectin were extracted using Extraction Kit (Comin, Suzhou, China). Finally, we obtained the cellulose, hemicellulose and protopectin contents using the colorimetric method. Each sample had three biological replications.

### Yeast one-hybrid assay

The sequence of *ClERF1* was inserted into the pGADT7 vector as the prey protein. The promoter sequence of *ClAux/IAA* and *ClEXT* containing ERF binding site was cloned into the pAbAi vector. The pAbAi plasmids were transformed into Y1H Gold yeast strain to determine the minimum inhibitory concentration of aureobasidin A (AbA), and then pGADT7-ClERF1 was transformed into bait strains. The transformed yeast was grown on SD/−Leu medium, and the interaction between ClERF1 and *ClAux/IAA* and *ClEXT* were detected on SD/−Leu medium with 70 ng/ml AbA.

## Results

### Parental evaluation of flesh firmness at different fruit developmental stages

**Fig. 1:**
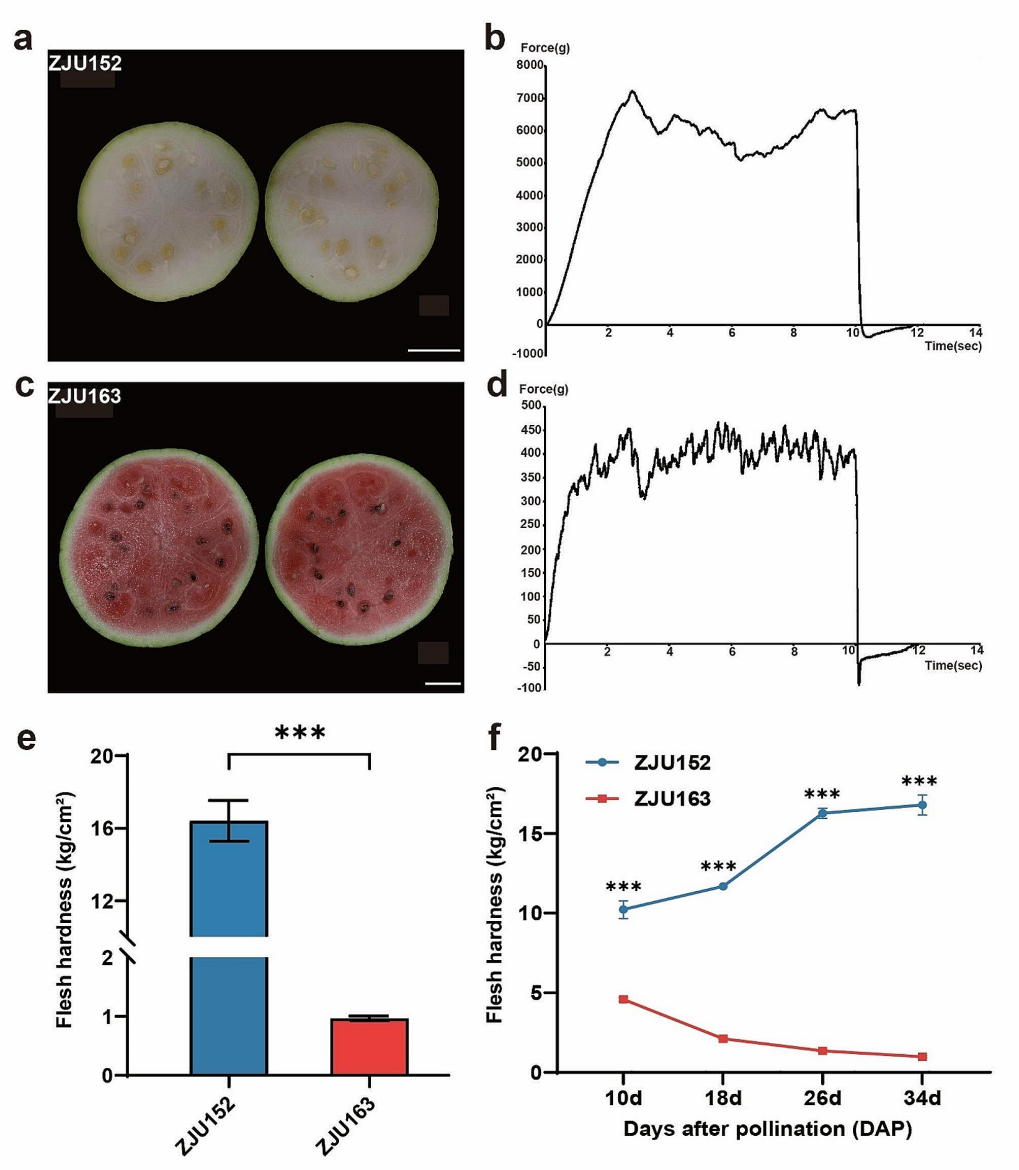
Flesh firmness phenotypes of the selected parents. **a** ZJU152 (*Citrullus mucosospermus*) watermelon fruit. Scale bars, 5 cm. **b** The force characteristic curve of ZJU152. **c** ZJU163 (*Citrullus lanatus*) watermelon fruit. Scale bars, 5 cm. **d** The force characteristic curve of ZJU163. **e** The flesh firmness of mature fruits of ZJU152 and ZJU163. **f** The variations in flesh firmness of ZJU152 and ZJU163 at 10, 18, 26, 34 DAP. *** *P* < 0.001

To investigate the inheritance pattern of the flesh firmness trait in watermelon, we developed a RIL gene mapping population by crossing hard-flesh and soft-flesh accessions. The male parent ZJU152 was the homozygous egusi seed watermelon with white flesh, and the female parent ZJU163 was the homozygous cultivated watermelon with red flesh (Fig. 1a, c). The phenotypes of the flesh firmness were determined by the force characteristic curves (Fig. 1b, d). To obtain an effective index for flesh firmness in watermelon, both center flesh firmness and edge flesh firmness were measured in two parents and derived F_6_ population, and the Pearson correlation coefficients between center and edge flesh firmness were calculated to confirm their relevance. There was a significant positive correlation between center flesh firmness and edge flesh firmness (Fig. S1), suggesting that center flesh firmness can be representative of flesh firmness of whole fruit, and can be used as a reliable indicator of flesh firmness.

The mature fruit samples between the two parental accessions ZJU152 and ZJU163 were measured using a texture analyser to assess their center flesh firmness. The result showed that the flesh firmness of the ZJU152 was 16.41 kg/cm^2^, while that of ZJU163 was 0.97 kg/cm^2^ (Fig. 1b, d, e), illustrating that ZJU152 was a hard-flesh line and ZJU163 was a soft-flesh line. Also, center flesh firmness was surveyed at four key developmental stages: 10, 18, 26 DAP and 34 DAP, which showed significant differences between all stages of two parents (Fig. 1f). During fruit developmental stages, the flesh firmness of ZJU152 increased rapidly in the early stages and remained stable after 26 DAP, and the minimum flesh firmness was measured at 10 DAP. In contrast, the flesh firmness of ZJU163 was consistently lower and decreased gradually, and the maximum firmness was measured at 10 DAP. Altogether, the flesh firmness of ZJU152 was significantly higher than the flesh firmness of ZJU163.

### Genetic and phenotypic analysis of flesh firmness using RIL populations

The average flesh firmness of F_1_ was 11.80 kg/cm^2^, which was between the parents (Fig. S2a), suggesting that the inheritance of the hard flesh was partially dominant. Furthermore, the variable of flesh firmness in F_6_ and F_8_ populations ranged from 1.96 kg/cm^2^ to 18.58 kg/cm^2^ and 0.98 kg/cm^2^ to 19.14 kg/cm^2^, respectively, and both showed an approximately normal distribution (Fig. S2a-b) and also performed a high correlation between two populations (Fig. S2c), which demonstrated that the flesh firmness trait was a quantitative trait controlled by multiple genes.

Genetic models for flesh firmness were calculated based on the phenotypic data of P1, P2, RIL (F_6_ and F_8_) populations. The optimal genetic models for flesh firmness of F_6_ and F_8_ population were calculated by SEA were MX2-DE-A and MX3-AI-A, respectively, according to the lowest AIC values and no significant parameter (*p* < 0.05) in the goodness-of-fit tests (Table S1; Table S2). The result further proved that the flesh firmness was a complex trait controlled by multiple genes.

### Primary mapping of candidate QTL for flesh firmness via BSA-seq

**Fig. 2:**
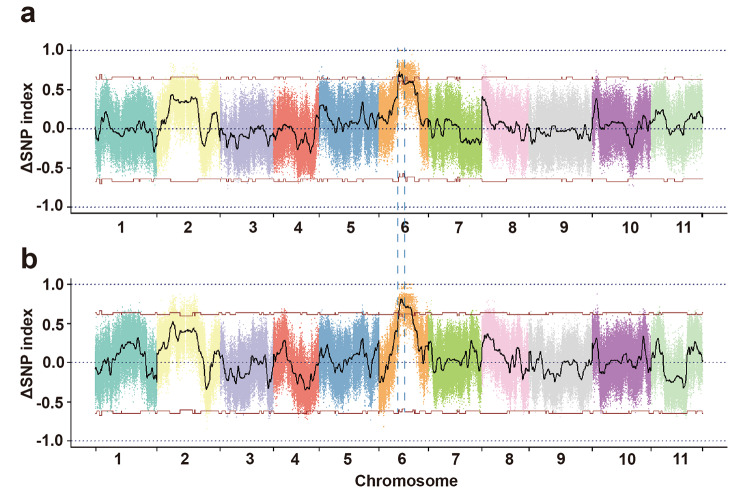
The BSA-seq analysis results of flesh firmness in the RIL populations. **a, b** Graph of ΔSNP index values used for the flesh firmness trait association analysis in F_6_ (a) and F_8_ (b) population. The x-axis indicates the 11 watermelon chromosomes, the y-axis represents the ΔSNP index. The black curve indicates the ΔSNP index. The red horizontal lines indicate the threshold lines of 99% confidence interval. The blue dotted lines represent the overlapping interval

To anchor the candidate locus for the watermelon flesh firmness, the primary mapping of QTL was first performed based on the BSA-seq by whole-genome resequencing of the two parents and the two pools with extreme phenotypes in the F_6_ and F_8_ populations. Among the 144 F_6_ individuals and 135 F_8_ individuals, 20 extreme phenotypic individuals with hard-flesh and 20 soft-flesh were selected to generate the F_6_-Hard-pool, F_6_-Soft-pool, F_8_-Hard-pool and F_8_-Soft-pool, respectively. Genomic DNA from these 20 high-firmness individuals and 20 low-firmness individuals were mixed equally. We obtained a total of 73.6 Gb of clean data with high quality (Table S3) by sequencing DNAs of two parental lines (ZJU152, ZJU163) and four pools (F_6_-Hard-pool, F_6_-Soft-pool, F_8_-Hard-pool and F_8_-Soft-pool). Ultimately, we obtained approximately 19.24, 13.39, 9.18, 10.37, 11.12 and 10.32 Gb of clean reads from ZJU152, ZJU163, F_6_-Hard-pool, F_6_-Soft-pool, F_8_-Hard-pool and F_8_-Soft-pool, respectively. Mapping the reads to the genome of *Citrullus lanatus* (Watermelon (97,103) v2) resulted in 98.54-99.09% mapping rates with 42.52×, 32.18×, 17.25×, 19.56×, 22.25× and 19.99× average depths for ZJU152, ZJU163, F_6_-Hard-pool, F_6_-Soft-pool, F_8_-Hard-pool and F_8_-Soft-pool (Table S3), respectively. In total, we obtained 591,033 and 590,120 homozygous SNPs between two parents using F_6_ and F_8_ populations. In order to identify the candidate interval associated with *ClFF* QTL, we calculated the ΔSNP index and then determined the candidate region by identifying the peak region where the ΔSNP index exceeded the threshold value. At the 99% significant level, we obtained the QTL (*ClFF*) located between the 12,076,742 to 15,073,822 bp interval on chromosome 6 in the F_6_ population, which overlapped with the interval obtained in the F_8_ population (Fig. 2a-b). The overlapping interval was referred to be the genetic region relevant to the flesh firmness.

### Fine mapping of *ClFF* gene associated with flesh firmness

To narrow down the genetic interval of *ClFF* QTL, we employed KASP markers in the candidate region. A total of 12 pair of KASP primer combinations were specifically developed in the primary mapped genomic region to screen for recombinants among F_6_ individuals, which were identified to be polymorphic between two parents. The *ClFF* QTL was then narrowed down to a 651 kb region between markers K4 and K5. To further fine map *ClFF* QTL, we genotyped F_6_ individuals using KASP primers designed between K4 and K5, and 3 critical recombinant plants were identified from them. Significant differences in flesh firmness were observed between F_6_-56 (considered as soft flesh) and F_6_-161 (considered as hard flesh) individuals, which were genotyped identically by the K15 and K16 markers.

**Fig. 3:**
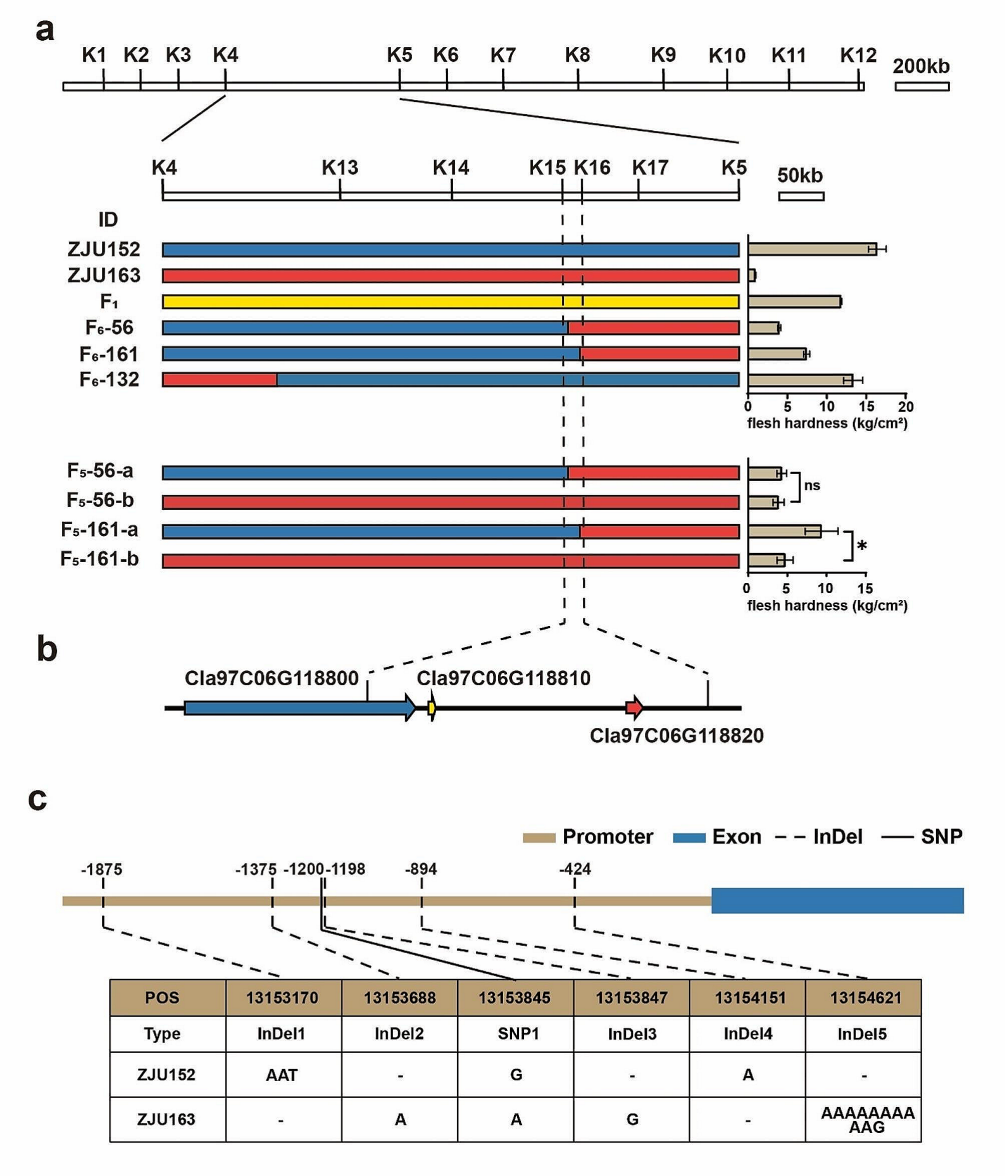
Fine mapping of *ClFF* gene associated with flesh firmness **a** Fine mapping of candidate region by analyzing F_6_ and F_5_ individuals with chromosome segment substitution using 17 KASP markers. A candidate region (15.5 kb in size) was identified between molecular markers K15 and K16 by further fine mapping of the recombinant individuals. Blue line indicates homozygous genotype as ZJU152, red line indicates homozygous genotype as ZJU163, yellow line indicates the heterozygous genotype. *P < 0.05, ns (non-significant difference). **b** Candidate genes in the target region. **c** The sequence variations in the promoter region of *Cla97C06G118820*.

Based on the genotypes of F_6_ recombinants, we further investigated the genotypes of the corresponding F_5_ lines. We identified two different genotypes in both F_5_-56 line and F_5_-161 line, and found no significant difference of flesh firmness between the two genotypes of F_5_-56. Meanwhile, significant differences of flesh firmness were discovered between two genotypes of F_5_-161 (F_5_-161-a and F_5_-161-b). Based on the phenotype of the recombinants, totally we used 17 KASP markers to do the fine mapping and placed the *ClFF* in a genomic region flanked by marker K15 and K16, which was 13143.2-13158.7 kb (Table S4). Finally, we anchored a 15.5 kb target region on chromosome 6 in *Citrullus lanatus* (Watermelon (97,103) v2) genome that contained three genes (*Cla97C06G118800, Cla97C06G118810, Cla97C06G118820*) (Fig. 3a-b).

To further identify the candidate gene for *ClFF*, we analyzed the variations of these three genes between two parents. Indeed, among the three genes in the target region, there was no sequence variation in the coding sequences between two parents. We also analyzed the variants in the promoter region of these three genes. The promoter of *Cla97C06G118800* has a SNP variation, which was not located in the fine-mapping interval and did not co-segregate with the phenotype, and no variation in the promoter of *Cla97C06G118810* was observed. Whereas, several variations were found in the promoter region between two parents of *Cla97C06G118820*, which contained one SNP and five InDels (Fig. 3c). Therefore, *Cla97C06G118820* was speculated as the most likely candidate gene of *ClFF* designated as *ClERF1*, which was predicted to encode an ethylene-responsive transcription factor 1.

### Variations in the promoter and haplotype analysis of *ClERF1* gene

**Fig. 4:**
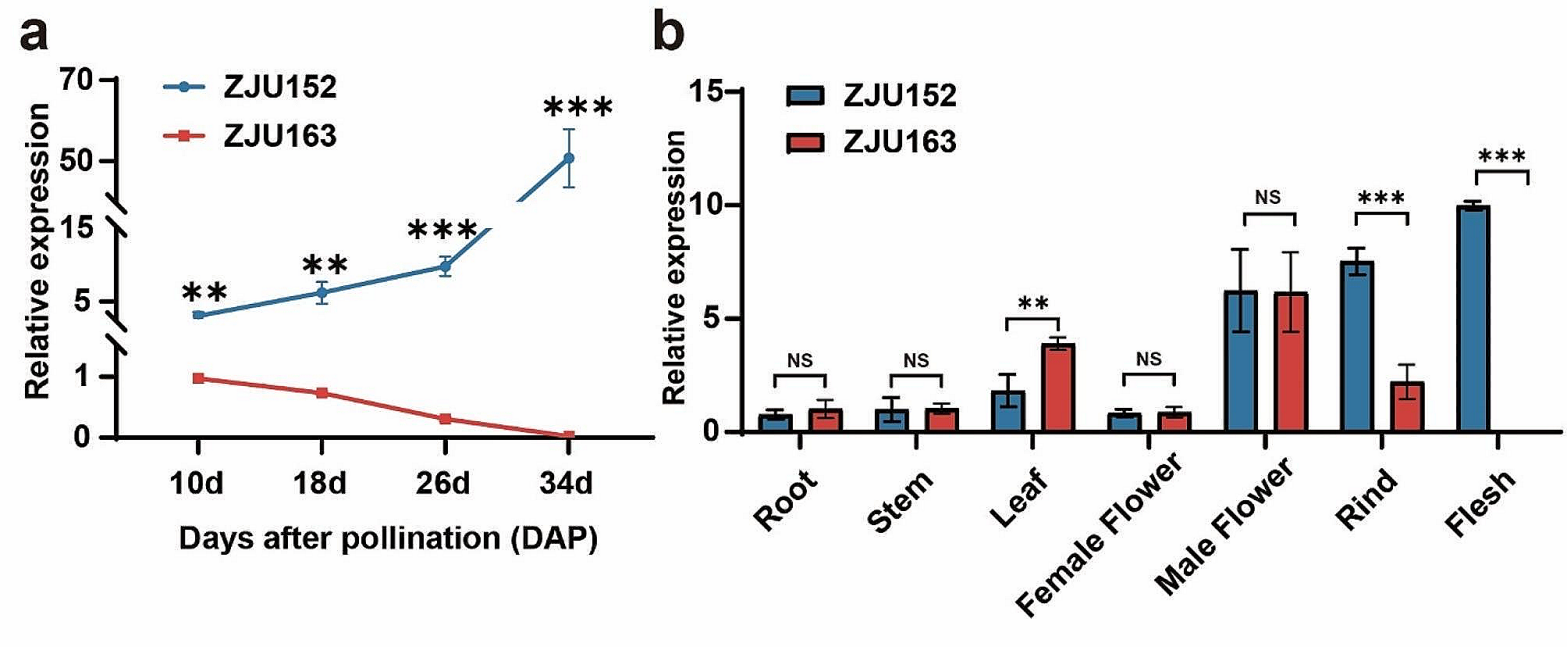
Expression levels of the *ClERF1* in ZJU152 and ZJU163. **a** Relative expression of *ClERF1* between the flesh of ZJU152 and ZJU163 at four key developmental stages. **b** Tissue-specific expression patterns of *ClERF1* in ZJU152 and ZJU163. **P < 0.01, ***P < 0.001, ns (non-significant difference)

Then we further checked the expression levels of *ClERF1* in the flesh between two parents at key developmental stages to determine whether the variations in the promoter affected the expression levels of *ClERF1*. The results indicated that *ClERF1* showed a consistent difference of expression levels in the flesh between ZJU152 and ZJU163 at four key developmental stages, and significantly lower expression levels were observed in ZJU163 (Fig. 4a). Additionally, we evaluated the *ClERF1* expression in several tissues of parents, and found that it was expressed mainly in male flowers, rind and flesh. Moreover, the expression levels of ZJU163 in the rind and flesh were significantly lower than that of ZJU152 (Fig. 4b). The expression pattern of *ClERF1* between two parents was consistent with the variations of flesh firmness at four key developmental stages (Figs. 1f and 4a), indicating that the high expression profiling of *ClERF1* might be associated with the variations of flesh firmness. Furthermore, we hypothesized that these variations in the promoter of *ClERF1* would result in increased promoter activity in ZJU152, leading to an increase in its expression. As expected, dual-luciferase assay in tobacco showed that the ZJU152 type promoter of *ClERF1* had significantly higher relative activity than the ZJU163 type (Fig. S3a-b). The GUS transient assay in tobacco leaves also showed that the *ClERF1* promoter activity in ZJU 152 was significantly higher than that of ZJU163 (Fig. S3c-d). Thus, we validated that the expression level of *ClERF1* is regulated by the promoter variations, which may in turn affect the variation in flesh firmness.

**Fig. 5:**
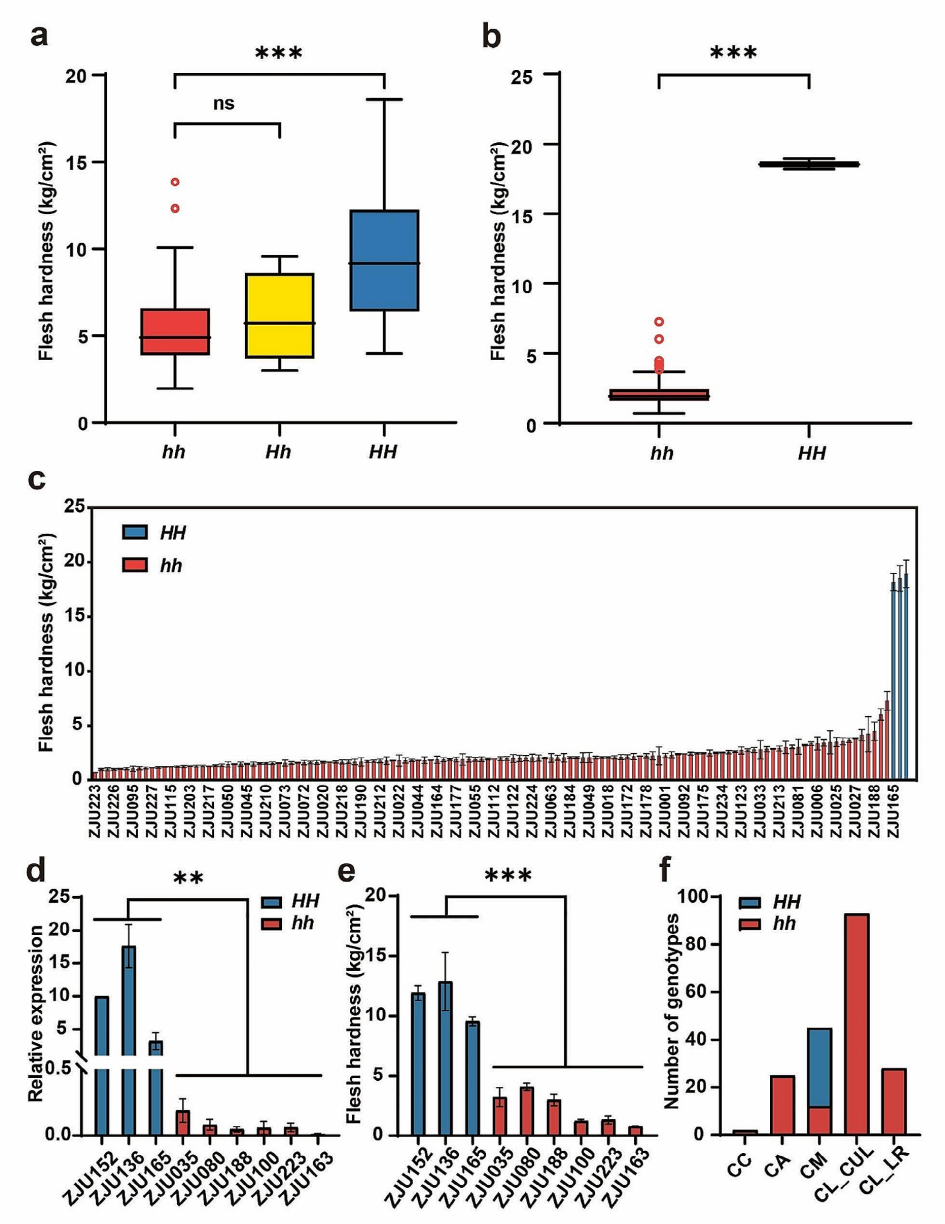
Associations between flesh firmness variations and the allelic distributions of *ClERF1*. **a, b** Association analysis between *ClERF1* genotypes based on InDel5 and flesh firmness in 144 F_6_ individuals (a) and 126 germplasm accessions (b). *HH* indicates homozygous type of ZJU152, *hh* indicates homozygous type of ZJU163 and *Hh* indicates heterozygous genotypes. **c** The flesh firmness and genotype of the 126 germplasm accessions. **d, e** Expression analysis of *ClERF1* (d) and flesh firmness (e) in nine accessions with different genotypes. **f** Number of genotypes in different species. CC, *C. colocynthis*; CA, *C. amarus*; CM, *C. mucosospermus*; CL_CUL, *C. lanatus* cultivar; CL_LR, *C. lanatus* landrace. **P < 0.01, ***P < 0.001, ns (non-significant difference)

To further study the variations in the promoter of *ClERF1* and its relevance to flesh firmness, a KASP marker based on the 11 bp deletion in the ZJU152, InDel5, was developed for genotyping. Among 144 individuals from F_6_ population, we found that the flesh firmness in genotype *hh* (homozygous genotype with ZJU163) was significantly lower than *HH* (homozygous genotype with ZJU152), and the flesh firmness of heterozygous genotype *Hh* showed no significant difference from that of *hh* (Fig. 5a; Table S5). We also observed the same results among 135 individuals from F_8_ population (Fig. S2d; Table S6). Regression analysis showed that this marker was responsible for 30.1% and 29.3% of flesh hardness variation in F_6_ and F_8_ population, respectively. Consistent with the RIL populations, the flesh firmness of the *hh* genotype was significantly lower than that of *HH* among the 126 germplasm accessions (Fig. 5b-c; Table S7). Additionally, we checked the *ClERF1* expression in some germplasm accessions with different genotypes. It was found that expression of *ClERF1* were significantly lower in the accessions with *hh* genotype (ZJU035, ZJU080, ZJU188, ZJU100, ZJU223, ZJU163) than in the accessions with *HH* genotype (ZJU136, ZJU152, ZJU165) (Fig. 5d). The flesh firmness of these two grouping accessions also showed the consistent difference, with the *HH* genotype accessions showing significantly higher flesh firmness than the *hh* genotype accessions (Fig. 5e).

Interestingly, the hard fleshes with *HH* genotypes were only observed in the wild relative of *C. mucosospermus* accessions (ZJU136, ZJU152, ZJU165), and not even in the wild relative of *C. amarus* accessions, suggesting that the *HH* genotype might only contribute to the hard flesh phenotype in *C. mucosospermus* (Table S7). Moreover, we also investigated the allelic variations of more wild relative watermelon accessions to further verify whether *HH* genotype only existed in *C. mucosospermus* accessions (Table S8). In total, 193 watermelon accessions were used to investigate the allelic variations, and it was observed that the *HH* genotype was only present in the wild relative of *C. mucosospermus* accessions among these watermelon accessions. However, not all *C. mucosospermus* accessions were *HH* genotypes, and 26.67% (12/45) *C. mucosospermus* accessions had *hh* genotypes (Fig. 5f), indicating that *C. mucosospermus* might have mutated during evolution, while some *C. mucosospermus* accessions remained original genotypes.

These data strongly suggested that variations in the promoter lead to the differential expression levels of *ClERF1*, which played an important role in regulating flesh firmness. Collectively, these results strongly supported *ClERF1* as a candidate gene relevant to watermelon flesh firmness.

### *ClAux/IAA* and *ClEXT* are supposed to be targeted by ClERF1

**Fig. 6:**
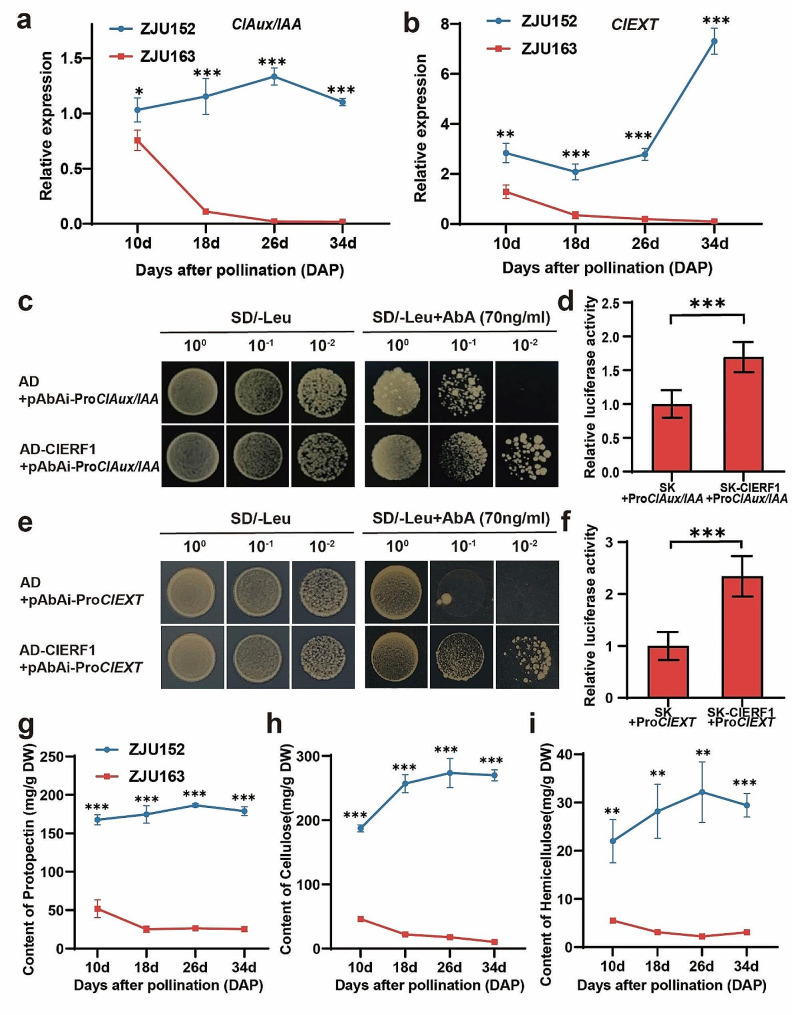
ClERF1 can bind to the promoters of *ClAux/IAA* and *ClEXT* to promote their expression. **a, b** Relative expression of *ClAux/IAA* (a) and *ClEXT* (b) between the flesh of ZJU152 and ZJU163 at four key developmental stages. **c-f** Y1H assay suggested that ClERF1 can bind to the promoters of *ClAux/IAA* (c) and *ClEXT* (e). Dual-luciferase assay indicated that ClERF1 can activate the expressions of *ClAux/IAA* (d) and *ClEXT* (f). **g-i** Content of protopectin (g), cellulose (h) and hemicellulose (i) in ZJU152 and ZJU163 at four key developmental stages. *P < 0.05, **P < 0.01, ***P < 0.001

Prior study revealed that the auxin responsive protein (*ClAux/IAA*) was related to the flesh hardness in watermelon, and its expression level was high positively correlated with *ClERF1* [24, 25]. In addition, *Cla97C06G118800* annotated as an exostosin family protein (*ClEXT*) was reported as a hub gene associated with cell wall biosynthesis and responsible for watermelon flesh firmness, which also performed a significant difference in expression levels between two genotypes with significant different in flesh firmness [46]. In this study, we further checked the expressions of *ClAux/IAA* and *ClEXT* in the flesh between two parents at key developmental stages. The results indicated that the expressions of *ClAux/IAA* and *ClEXT* in ZJU163 were significantly lower than that in ZJU152 at four key developmental stages, and also displayed consistent expression pattern with *ClERF1* (Figs. 4a and 6a-b). We also checked the expression levels of *ClAux/IAA* and *ClEXT* in some germplasm accessions, and found that the expression levels of hard-flesh group accessions (ZJU136, ZJU152, ZJU165) were significantly higher than that of soft-flesh group accessions (ZJU035, ZJU080, ZJU188, ZJU100, ZJU223, ZJU163), which were also similar to the expression pattern of *ClERF1* in these germplasm accessions (Figs. 5d-e and S4a-b). Therefore, we speculated that *ClAux/IAA* and *ClEXT* may be targeted by *ClERF1* involving in cell wall biosynthesis and fruit ripening.

In this study, we found that there were ERF-binding sites in the promoters of *ClAux/IAA* and *ClEXT*, indicating that ERF might activate the expression levels of *ClAux/IAA* and *ClEXT* by binding to their promoters. To determine whether ClERF1 could bind to their promoters, we conducted Y1H and dual-luciferase assays, and found that ClERF1 was indeed able to target the promoters of *ClAux/IAA* and *ClEXT* and activated their expressions (Fig. 6c-f). In summary, these results indicted that ClERF1 directly bound the promoters of *ClAux/IAA* and *ClEXT* and positively regulated their expressions.

We also measured the cell wall components contents to provide biochemical insights into whether *ClERF1* influenced the fruit ripening and cell wall biosynthesis to cause the variations in flesh firmness. At four key developmental stages, cellulose, pectin and hemicellulose contents were measured. The results indicated that the contents of cell wall components in ZJU163 were significantly lower than that in ZJU152 during fruit development, which may have a significant impact on the variation of flesh firmness (Fig. 6g-i). In conclusion, these results provide molecular and biochemical evidences that ClERF1 might participate in cell wall biosynthesis and fruit ripening process by directly targeting the *ClAux/IAA* and *ClEXT* promoters leading to the flesh firmness variations in watermelon.

## Discussion

Fruit softening is a complex biological process which is controlled by interactions between genetic factors, plant hormones and environment [47–51]. Changes in firmness of flesh are one of the major characteristics during fruit development and maturity, and are known to affect fruits taste, storability, transportability, and shelf-life. The fruit flesh firmness is a major breeding target of fruit crops like watermelon. Elucidating the genetic and molecular regulatory mechanisms and identifying key genetic factors of flesh firmness in watermelon will provide theoretical and technical support for the improvement of watermelon quality. However, most researches of flesh firmness in watermelon focused on the comparative transcriptome analysis [34, 46, 47], and no attempts were reported to fine-map the flesh firmness associated genes. In the present study, we identified a *ClFF* locus on chromosome 6 which controls the flesh firmness in watermelon using BSA-seq analysis and RIL populations crossed from hard-flesh and soft-flesh parents. Through fine mapping, *ClFF* was successfully narrowed down to a 15.5 kb genomic region containing 3 genes (Fig. 3a-b). Finally, *ClERF1* encoding an ethylene responsive factor 1 possessed several variations in the promoter, and was identified to be the major gene associated with flesh firmness through combinatorial expression and haplotype analysis.

Furthermore, the *HH* genotype of *ClERF1* might only exist in the wild relative of *C. mucosospermus*, which has extremely hard flesh (flesh firmness > 18 kg/cm^2^) (Table S7). We also evaluated the genotypes in other wild accessions, and found that the *HH* genotype was observed only in *C. mucosospermus* and 73.3% (33/45) *C. mucosospermus* had *HH* genotypes (Fig. 5f, Table S8). These results indicated that *C. mucosospermus* might have mutated during evolution and the *HH* genotype might only contribute to the hard-flesh phenotype in *C. mucosospermus*. Phylogenetic and population structure inferences revealed that *C. mucosospermus* and *C. lanatus* derived from the same ancestor and were probably domesticated for different purposes: one for seed consumption and the other for fruit flesh [18]. Due to the different purposes of domestication, the hard-flesh allelic variations might be lost in most cultivated watermelons (*C. lanatus*). Moreover, the variations of *ClERF1* were hardly related to the difference in flesh firmness among natural *C. lanatus* accessions, as no *HH* genotype was identified in any of the tested *C. lanatus* lines in this study (Fig. 5f, Table S7). The exceptional phenomenon also suggests that the loci/genes of *C. mucosospermus* had not been introduced into watermelon cultivated species *C. lanatus* through wide-hybridization. It is also necessary to further identify the other QTLs/genes that may be associated with the variations of different flesh firmness among *C. lanatus* accessions, which would be useful in the future improvement of flesh firmness without compromising crispy taste in *C. lanatus*. *C. mucosospermus* exhibited wider genetic variations and phenotypic traits [52, 53], and several *C. mucosospermus* germplasm lines selected for use in watermelon breeding program to enhance disease resistance [54, 55]. *C. mucosospermus* also provided useful genetics variations for improvement of fruit qualitative traits, such as fruit size, shape, colour and taste [52]. In this study, the hard-flesh gene is valuable in genetic improvement of watermelon cultivars for long transportation and storability through wide hybridization.

ERFs belong to the AP2/ERF superfamily and play important roles in fruit ripening and softening as transcription factors regulating ethylene biosynthesis and signal transduction [28, 56–59]. Extensive studies have shown the possible involvement of ERFs in fruit ripening and softening in climacteric fruits [12, 14, 31, 60, 61]. Some previous studies also provided evidence that ethylene is involved in the ripening of non-climacteric fruits such as strawberry and citrus [62, 63]. Although watermelon as a non-climacteric fruit, the regulation of ethylene on fruit ripening and flesh firmness during fruit development cannot be ignored, and some ERFs have been reported associated with fruit development [64, 65]. Through comparative transcriptome analysis of two cultivars with significant differences in flesh firmness of watermelon, two *ERF* genes were identified as candidate genes, and significant difference in expression levels were observed in two cultivars, which may regulate the cell wall biosynthesis genes involve in flesh firmness changes in watermelon fruits [46]. Interestingly, *ClERF1* (*Cla97C06G118810*) identified in this study was *Cla004120*, one of these two *ERF* genes annotated in *Citrullus lanatus* (Watermelon (97,103) v1). The expression levels of *Cla004120* also differed significantly between two genotypes with significant difference in flesh firmness (Figs. 1f and 4a), further confirming that *ClERF1* is a major gene responsible for flesh firmness in watermelon.

Accumulating evidences have confirmed that ERFs were associated with fruit ripening and cell wall biosynthesis [66–69]. DkERF8 and DkERF16 activated cell-wall-modifying genes *DkXTH11* and *DkEXP4*, respectively, to participate in persimmon fruit ripening [33]. It was discovered that CpERF9 regulates papaya fruit ripening by binding to the promoters of *CpPME1/2* and *CpPG5* to suppress their transcription [70]. Besides, the auxin-mediated fruit ripening also play an important role in flesh firmness. The expression levels of *PpPG* and *Ppβ-GAL* genes related to cell wall softening and *PpACS1* genes linked to ethylene synthesis were found to be reduced by NAA treatment [71]. In peach, PpIAA1 and PpERF4 were found to form a positive feedback loop to regulate peach fruit ripening by integrating auxin and ethylene signals. Furthermore, *PpIAA1* overexpression in tomato accelerated fruit ripening and shortened the fruit shelf life [32]. Down-regulation of DR12, an auxin-response-factor homolog, enhanced the fruit firmness in the tomato [72]. It is reported that the auxin responsive protein (*ClAux/IAA*) was related to the flesh hardness in watermelon, and its expression level was high positively correlated with *ClERF1* [24, 25]. *Ethylene-responsive factor 4* was reported to be associated with the rind hardness trait in watermelon, which is related to the regulation of cell wall biosynthesis and degradation-associated genes [20]. The phylogenetic analysis of ERF superfamily performed by Liao et al., (2020) showed that *ClERF1* and *ClERF4* were physically close and both belonged to the member of the group III ERFs. So we speculated that the regulation pattern of *ClERF1* in flesh firmness may be similar to *ClERF4* in rind hardness, which may have important effects in cell wall biosynthesis and modification like other group III ERF members [28, 31, 69, 73, 74]. *ClEXT* was a putative glycosyltransferase, and its homolog genes of *Arabidopsis* were reported to belong to the glycosyltransferase family 47, members of which were predicted to be membrane-bound glycosyltransferase involved in cell wall biosynthesis. The glycosyltransferase family 47 member XGD1 was found to possess XGA xylosyltransferase activity, which was involved in pectin biosynthesis in *Arabidopsis* [75]. In this study, Y1H and DUAL-LUC assays indicated that ClERF1 bound to the promoters of *ClAux/IAA* and *ClEXT* and activated their expressions (Fig. 6c-f), which further supported that ClERF1 participate in cell wall biosynthesis and fruit ripening process to regulate flesh firmness.

## Conclusions

In this study we reported the potential candidate gene *ClERF1* for variations in flesh firmness via fine mapping, filling a gap in fine mapping studies of flesh firmness in watermelon. ClERF1 directly bound the promoters of *auxin-responsive protein* (*ClAux/IAA*) and *exostosin family protein* (*ClEXT*) and positively regulated their expressions influencing fruit ripening and cell wall biosynthesis, which led to the variations of flesh firmness in watermelon. Obviously, flesh firmness is largely accounted for many desirable commercial traits like storability and shelf-life in fresh fruit crops. This finding of flesh-firmness gene will facilitate the marker-assisted precision breeding of watermelon flesh-firmness improvement.

### Electronic supplementary material

Below is the link to the electronic supplementary material.

Supplementary Material 1

Supplementary Material 2

## Data Availability

No datasets were generated or analysed during the current study.
