# Supplementary material for: Promoter variations of ClERF1 gene determines flesh firmness in watermelon
Source: BMC Plant Biol. 2024 Apr 16;24:290. doi: 10.1186/s12870-024-05000-z (PMC11020897; doi:10.1186/s12870-024-05000-z)
Supplement: Supplementary file 2 — Supplementary Material 2 [file 12870_2024_5000_MOESM2_ESM.docx]

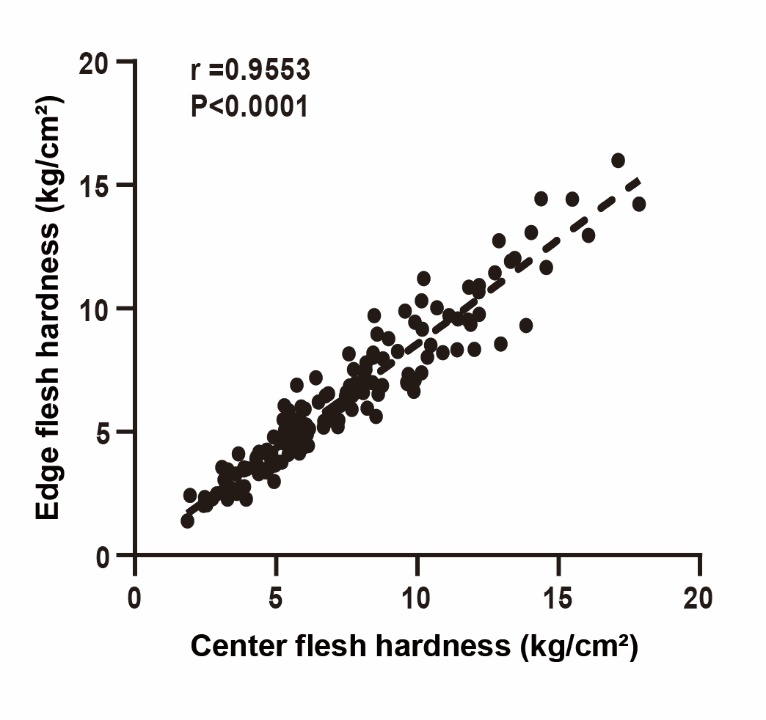


**Fig. S1.** The correlation between center flesh hardness and edge flesh hardness in F_6_ population.


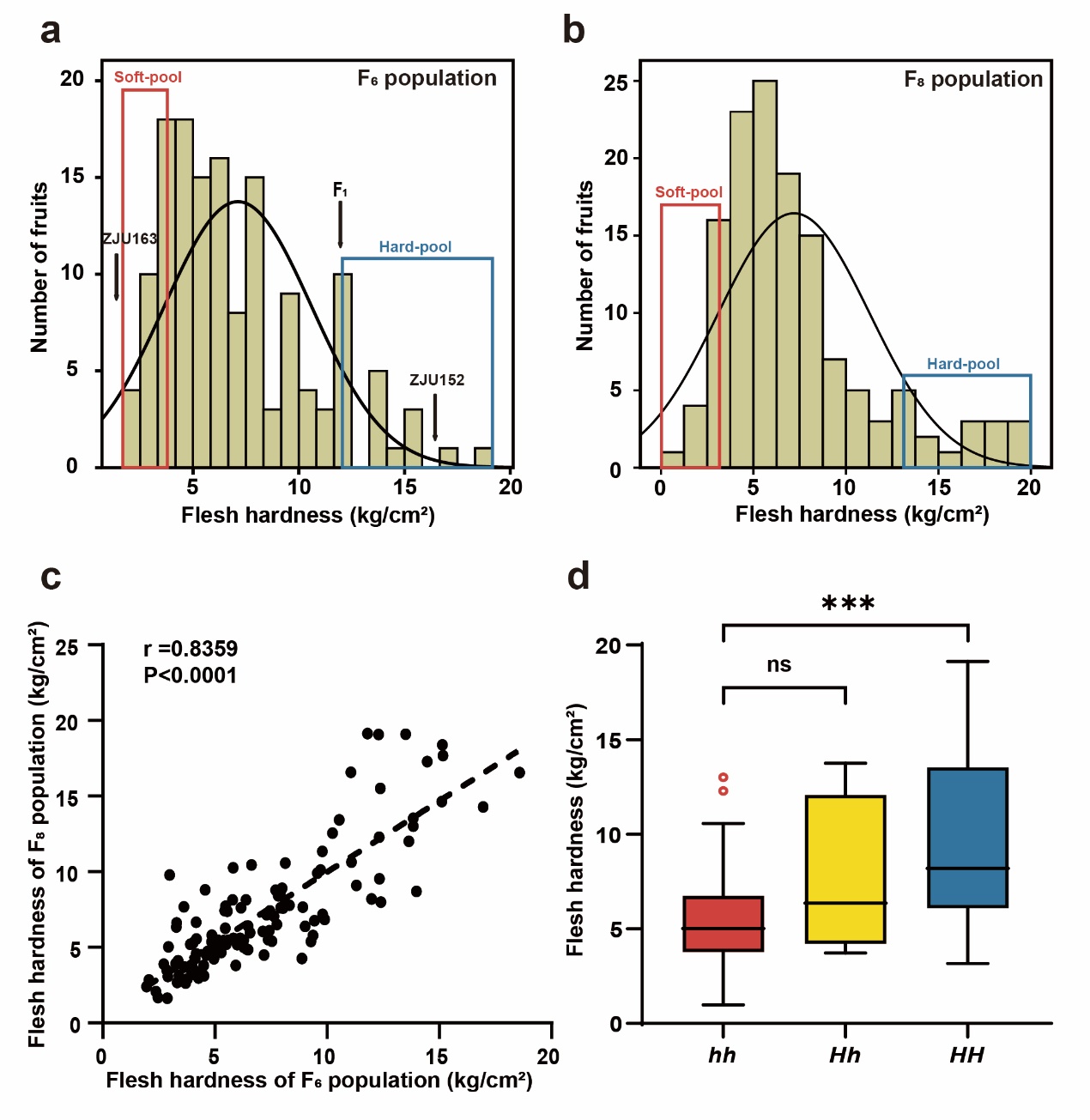


**Fig. S2.** Associations between the flesh hardness of F_6_ population and F_8_ population. **a** Frequency distribution of flesh hardness in F_6_ population. The three arrows indicate the flesh hardness of the ZJU163 and ZJU152 parental lines and F_1_. **b** Frequency distribution of flesh hardness in F_8_ population. The red and blue boxes indicate the 20 soft-flesh individuals that were used to make the Soft-pool, and the 20 hard-flesh individuals used to make the Hard-pool. **c** The correlation between flesh hardness of F_6_ population and flesh hardness of F_8_ population. **d** Association analysis between *ClERF1* genotypes based on InDel5 and flesh hardness in 135 F_8_ individuals. *HH* indicates homozygous type of ZJU152, *hh* indicates homozygous type of ZJU163 and *Hh* indicates heterozygous genotypes. ***(*P* < 0.001) indicates significant difference by Student’s T-test, while ns shows non-significant difference.


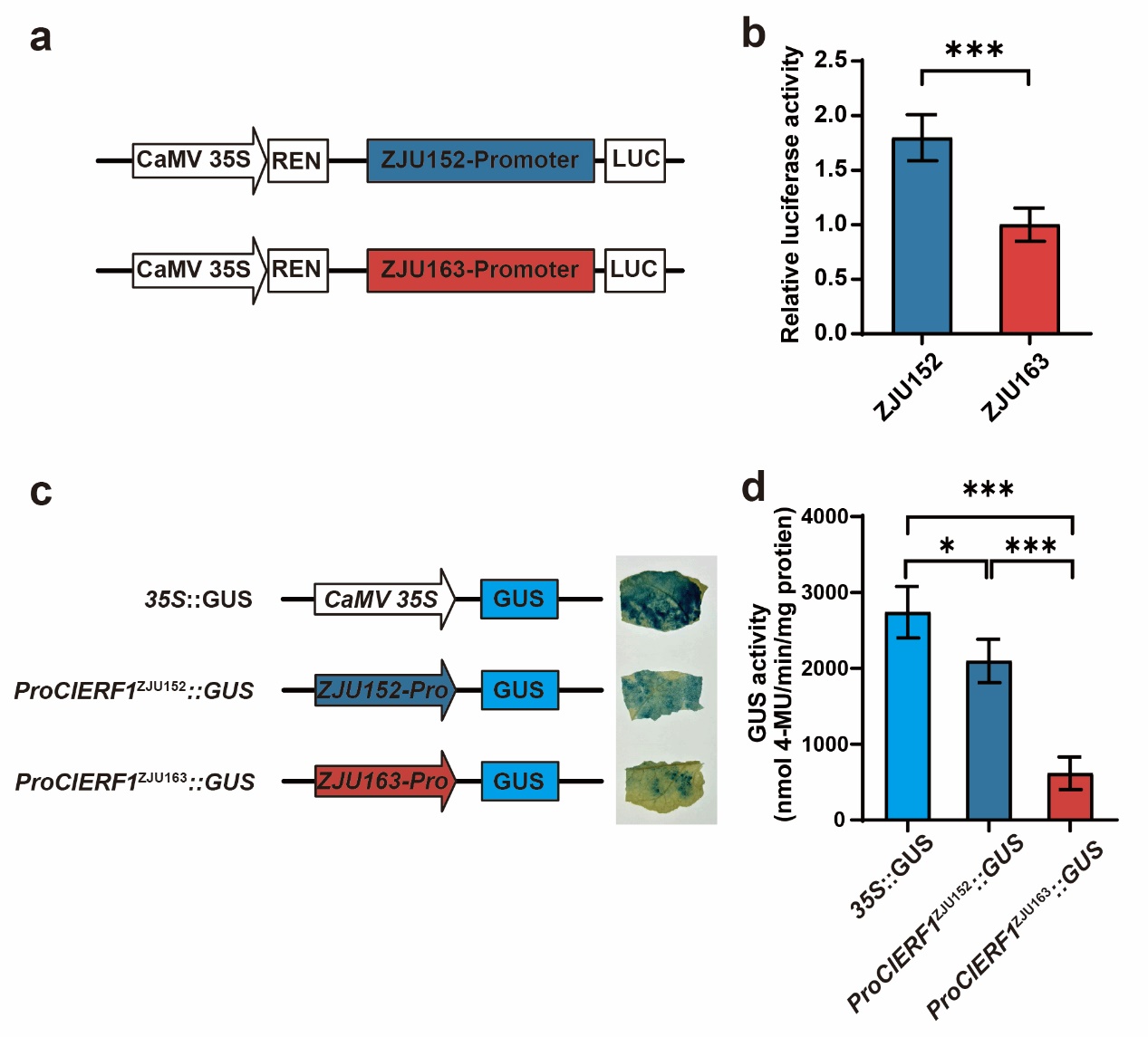


**Fig. S3.** Promoter activity of *ClERF1* between ZJU152 and ZJU163. **a** Schematic diagram of constructs for dual-luciferase assay. **b** Promoter activity of ZJU152 and ZJU163. The relative value of LUC/REN indicates promoter activity. The ratio of LUC/REN with ZJU163 was set to 1 for normalization. **c** Schematic diagram of constructs for GUS fusion expression vector**. d** GUS activity of the *ClERF1* promoter between ZJU152 and ZJU163 in tobacco leaves. *(*P*<0.05) and ***(*P* < 0.001) indicates significant difference by Student’s T-test.


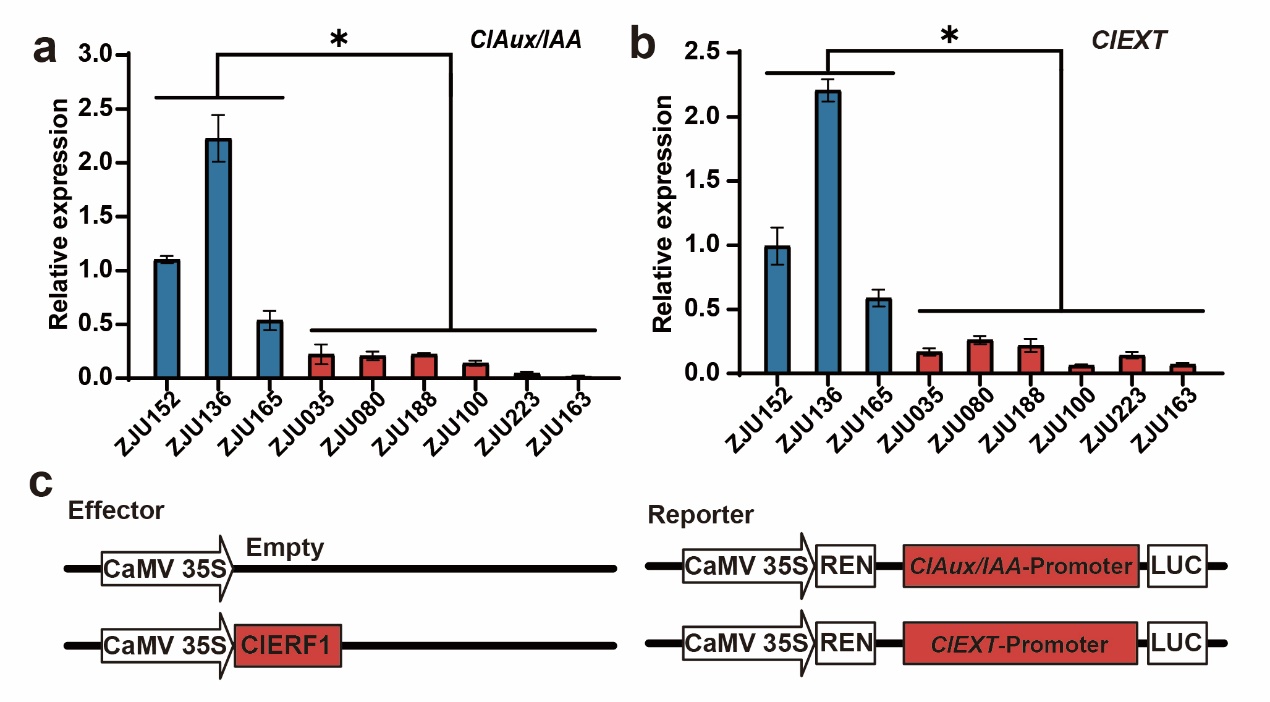


**Fig. S4.** Expression analysis of *ClAux/IAA* and *ClEXT* in a panel of germplasm accessions. **a, b** The relative expression of *ClAux/IAA* (a) and *ClEXT* (b) in nine accessions with different flesh firmness. **c** Schematic diagram of the effector and reporter vectors for dual-luciferase assays.
